# Identification of Pulmonary Infections With Porcine Rotavirus A in Pigs With Respiratory Disease

**DOI:** 10.3389/fvets.2022.918736

**Published:** 2022-06-23

**Authors:** April Nelsen, Kelly M. Lager, Judith Stasko, Eric Nelson, Chun-Ming Lin, Ben M. Hause

**Affiliations:** ^1^Animal Disease Research and Diagnostic Laboratory, Department of Veterinary and Biomedical Sciences, South Dakota State University, Brookings, SD, United States; ^2^National Animal Disease Center, USDA Agricultural Research Service, Ames, IA, United States

**Keywords:** rotavirus A, porcine respiratory disease complex, interstitial pneumonia, enteritis, extraintestinal rotavirus

## Abstract

While rotavirus (RV) is primarily known to cause gastroenteritis in many animals, several epidemiological studies have shown concurrent respiratory symptoms with fecal and nasal virus shedding. However, respiratory RV infections have rarely been investigated. By screening clinical samples submitted for diagnostic testing, porcine rotavirus A (RVA) was detected by quantitative reverse transcription PCR (qRT-PCR) in 28 out of 91 (30.8%) lungs obtained from conventionally reared pigs with respiratory signs. Among the positive cases, intensive RVA signals were mainly localized in alveolar macrophages (*n* = 3) and bronchiolar epithelial cells (*n* = 1) by RNAscope^®^ *in situ* hybridization (ISH). The signals of RVA in bronchiolar epithelial cells were verified by ISH with different probes, immunohistochemistry, and transmission electron microscopy. Furthermore, additional cases with RVA ISH-positive signals in alveolar macrophages (*n* = 9) and bronchial epithelial cells (*n* = 1) were identified by screening 120 archived formalin-fixed and paraffin-embedded lung samples using tissue microarrays. Overall, our study showed a high frequency of RVA detection in lungs from conventional pigs with respiratory disease. Further research is needed to determine if RVA infection in the respiratory epithelium correlates with nasal shedding of rotavirus and its contribution to respiratory disease.

## Introduction

Rotaviruses (RVs) are members of the *Reoviridae* family with a genome comprised of 11 linear double-stranded RNA segments that code for six structural proteins (VP1-4, 6, and 7) and six non-structural proteins (NSP 1–6). The conserved structural protein VP6 is highly immunogenic and is the major diagnostic target for RVs (1). Rotavirus is a significant global cause of acute enteritis in young children and animals, including neonatal and nursing piglets (2, 3). While 10 groups of RVs have been described, group A RVs (RVA) are the best studied as they are found in a wide array of species including pigs, humans, cattle, and poultry (4). It is well known that RVs infect mature enterocytes in the small intestine leading to disruption of the intestinal mucosal barrier and the development of diarrhea (2, 5). While RV principally replicates in intestinal epithelial cells, several studies have detected RV in serum and multiple visceral organs, suggesting viremia (5–11). In humans, evolving research shows RV infection has systemic effects, where extraintestinal RV antigenemia has been reported in numerous studies of children with a variety of clinical signs, including respiratory disease (12, 13).

Knowledge regarding respiratory infection of RVs is still limited. Rotaviruses have been detected in nasopharyngeal secretions in infants with respiratory illness, as well as neonatal piglets in experimental settings (6, 11, 12). In addition to enteritis, piglets orally inoculated with porcine RVA subtypes G9P [23] and G9P [7] also displayed interstitial pneumonia (14). Similarly, inoculation of pigs with a reassortant bovine RV resulted in enteric disease with RV being detected in the serum, lungs, and other organs by PCR and immunofluorescence assays (15). In these studies, RV antigen was mainly detected in enterocytes but also sporadically detected in the pneumocytes and lymphoid cells in the mesenteric lymph node, lungs, liver and choroid plexus. Furthermore, neonatal germ-free piglets that were inoculated either orally or intranasally with the human RVA strain Wa developed diarrhea and viremia and shed virus both nasally and rectally (6).

In our laboratory, routine metagenomic sequencing of diagnostic samples frequently detected RV in the lungs of pigs with respiratory disease. Diagnostic testing of respiratory tissues for RV is rarely performed given RV is not recognized as a primary respiratory pathogen. To date, there is no definitive tissue-based evidence demonstrating that RV can replicate in and damage lung tissues. The aims of the present study were to determine the prevalence of RV in lungs obtained from pigs with respiratory disease, and to characterize RV lung tissue distribution and its possible role in porcine respiratory disease complex (PRDC) pathogenesis.

## Materials and Methods

### Experimental Design

This study used two sampling strategies to assess the prevalence and histopathology of RVA in respiratory tissue. The first was to use frozen tissue homogenates (*n* = 91) retained from porcine respiratory diagnostic samples submitted to the South Dakota State University Animal Disease Research and Diagnostic Laboratory (SDSU ADRDL) between October 2020 and January 2021. These independent submissions stored at −80°C were tested for RVA by quantitative RT-PCR (qRT-PCR). Case data, including pig age, clinical signs, necropsy reports, and all client-requested diagnostic testing results were recorded. Pig age groups were determined as fetuses <1-day, suckling pigs (1 day−3 weeks), nursery pigs (3–8 weeks), grow-finish pigs (8–25 weeks), mature pigs (>25 weeks), and unknown. Subsequently, the RVA qRT-PCR-positive cases with formalin-fixed paraffin-embedded (FFPE) lung tissue blocks were selected for histopathology. Tissue distribution of RVA genomes and antigens were detected by *in situ* hybridization (ISH) and immunohistochemistry (IHC), respectively. Transmission electron microscopy (TEM) was applied to visualize the viral particles in respiratory epithelial cells positive for RVA by histopathology.

In the second approach, to rapidly screen a large number of samples, a tissue microarray (TMA) was used for high throughput screening as described previously (16). Retrospectively, 20 tissue blocks collected from nursing piglets with rotaviral enteritis and high titer of fecal RV shedding, as diagnosed previously in our laboratory, and 100 randomly selected PRDC-affected cases with unknown rotaviral enteritis submitted from 2015 to 2021, were used for TMA construction. Tissue cores with a diameter of 5 mm were extracted from donor lung sample blocks and transferred to a recipient paraffin block by AMSBIO LLC (Cambridge, MA). In total, 6 TMA blocks, each included 20 lung cores, were constructed and sectioned. Forty three of the 100 randomly selected PRDC-affected cases had corresponding fresh tissues with qPCR results. Three of the 20 tissue blocks collected from nursing piglets with rotaviral enteritis had corresponding fresh tissues. TMA slides were stained by ISH and IHC for the screening of RVs as described above. Fresh lung and intestine tissues from these cases were not available.

### Quantitative Reverse-Transcription PCR

Nucleic acids were extracted from homogenized lung tissue with the QIAamp^®^ Viral RNA Mini Spin kit, per manufacturer's instructions. Quantitative RT-PCR was performed following a published protocol (17). The primers were combined with the sample and incubated for 5 min at 95°C to denature dsRNA and immediately placed on ice to allow primer binding to the linear single stranded RNA. Quantitative RT-PCR was performed using Fast Virus 1-step reagents (ThermoFisher) on an Applied Biosystems 7,500 FAST Real-Time PCR System with cycling conditions of 30 min at 50°C and an initial 30 s hot start at 95°C followed by 45 cycles of 15 s at 95°C and 60 s at 60°C. Quantitative RT-PCR results for intestine samples were generated by the ADRDL using a proprietary method (Table 1).

**Table 1:** Summary of the results of porcine rotavirus A (RVA) qRT-PCR-positive cases.

**Sample ID**	**Age (days)**	**qRT-PCR (Ct value)**		**ISH (cell type)**	**Histopathology** **(pneumonia)**
		**Lungs**	**Intestines**		
9265	5	30.3	NEG	NEG	-
10036	NA	24.2	NA	NA	NA
10058	56	25.5	NA	NEG	+
10164	NA	28.6	NEG	NA	NA
10372	35	27.2	24.9	NEG	-
10373	21	23.4	NA	NEG	+
10560	28	22.9	26.2	NEG	+
10566	12	27.5	NA	NEG	+
10615	NA	24.1	35.9	POS (Macrophage)	+
18062	NA	22.8	23.7	POS (Macrophage)	+
18115	7	26.9	NA	POS (Macrophage)	−
18302	NA	27.7	NA	NA	−
19474	28	26.0	22.4	NEG	+
19824	NA	27.6	NA	NA	NA
21026	28	25.8	NA	POS (Bronchiolar epithelium)	+
21515	14	28.7	38.3	NEG	+
21601	NA	26.7	NA	NEG	−
21690	28	24.8	NA	NEG	+
22560	NA	29.7	NA	NA	NA
22935	NA	24.9	28.4	NEG	+
23136	NA	31.1	NA	NEG	+
23243	21	30.2	NA	NEG	−
23473	NA	27.8	NA	NEG	+
25105	28	19.6	22.7	NEG	+
25391	NA	25.0	NEG	NEG	+
25485	NA	21.1	NA	NEG	+
159	3	29.9	NA	NA	NA
951	35	27.6	NA	NEG	+

*In total, 28 out of 91 porcine lung samples submitted for routine diagnosis were positive by qRT-PCR. Where available, paired intestine samples were tested. In situ hybridization was conducted using probes targeting the VP6 gene. The presence of pneumonia was determined in routine hematoxylin and eosin-stained lung section*.

*NEG, negative; POS, positive; NA, not available; -, no pneumonia noted; +, pneumonia noted*.

### *In Situ* Hybridization

*In situ* hybridization was performed per the manufacturer's instructions using RNAscope^®^ (Advanced Cell Diagnostics). A RVA probe targeting the VP6 gene was obtained from Advanced Cell Diagnostics using a previous design (18). A second RVA probe targeting the NSP3 gene was designed based on a consensus sequence derived from all swine rotavirus NSP3 sequences in Genbank. Both probes were commercialized (VP6 probe Part ID 447231, NSP3 probe Part ID1125871; Advanced Cell Diagnostics). Intestines obtained from RVA qRT-PCR-positive piglets were used as a positive control for the development, optimization, and verification of ISH and IHC assays. Intestine and lung tested negative by RVA qRT-PCR were used as negative controls.

### Immunohistochemistry

The protocol was carried out with the mouse specific HRP/DAB Detection IHC Kit-Micro-polymer (Dako), per the manufacturer's instructions with minor modifications. Antigen retrieval was conducted by heating in a microwave (1,100 W) for 2 min and 10 s in pH 6.0 citrate buffer. Subsequently, the slides were loaded onto a Dako Autostainer-Universal Staining System. Commercially-available anti-rotavirus capsid antibody 2B4, a monoclonal IgG_2B_ antibody against RVA VP6 (Santa Cruz Biotechnology, Dallas, TX) was used for IHC detection. Additionally, IHC was performed with the anti-dsRNA antibody, clone J2, a monoclonal IgG_2AK_ antibody that detects dsRNA (Millipore Sigma, Massachusetts, MA). Anti-dsRNA binds to dsRNA 40-bp or longer, independent of their sequence (19).

### Transmission Electron Microscopy Examination

FFPE specimens positive by IHC and ISH were submitted to the National Animal Disease Center, USDA-Agricultural Research Service, for transmission electron microscopy. FFPE samples were used for EM. Identified areas of interest were taken from the FFPE block, melted and put in 2.5% glutaraldehyde in 0.1 M cacodylate buffer. They were post-fixed with 2% osmium, and then processed through graded alcohols, propylene oxide and Eponate 12 resin followed by a 48 hr polymerization. Thick sections (1 μm) were performed on select samples and a toluidine blue stain and basic fuchsin stain was applied. Polaroid photos were taken of these images, and the area of interest for thin sections was performed. A uranyl acetate and Reynold's lead stain were performed on the thin section before being examined with a ThermoFisher FEI Tecnai G^2^ BioTWIN electron microscope (FEI Co., Hillsboro, OR), and images were taken with Nanosprint12 camera (AMT Corp., Woburn, MA) (20).

## Results

### Detection of RVA in the Lungs and Intestines From Pigs With Respiratory Disease by qRT-PCR

Among the frozen lung homogenates (n = 91), 28 tested positive for RVA by qRT-PCR (Table 1). A high prevalence of RVA was detected in suckling (7/15, 46.7%) followed by nursery pigs (8/18, 44.4%) (Table 2). The cycle threshold (Ct) values of RVA detected in the lungs ranged from 19.6-31.1. Among cases with RVA-positive lung samples by qRT-PCR, 11 paired intestine diagnostic results were available. Eight out of the 11 paired samples had corresponding positive intestine samples.

**Table 2:** Prevalence of Rotavirus A (RVA) in lung tissue homogenates detected by qRT-PCR in different age groups.

**Age group**	**Number of samples** **(*n* = 91)**	**RVA-positive cases**
Fetuses (<1 day)	9	0 (0%)
Suckling Pigs (1 day−3 weeks)	15	7 (46.7%)
Nursery Pigs (3–8 weeks)	18	8 (44.4%)
Grow-Finish Pigs (8–25 weeks)	6	0 (0%)
Mature Pigs (>25 weeks)	1	0 (0%)
Unknown	42	13 (30.9%)

Of the 28 out of 91 lung homogenates that tested positive for rotavirus A by PCR, 17 (61%) showed pneumonia by histopathology. Five of the lung homogenates had no significant lesions and one had indications of “other respiratory illness”. Five homogenates did not have histopathology performed. Of the 63 lung homogenates that were PCR negative for rotavirus A, 33 (52%) were diagnosed with pneumonia by histopathology. Eight tissues were recorded as “other respiratory illness” and six had no significant lesions. Histopathology was not performed on 16 of the PCR negative tissues.

### Localization of RVA Genomes in the Lungs Using ISH

**Figure 1:**
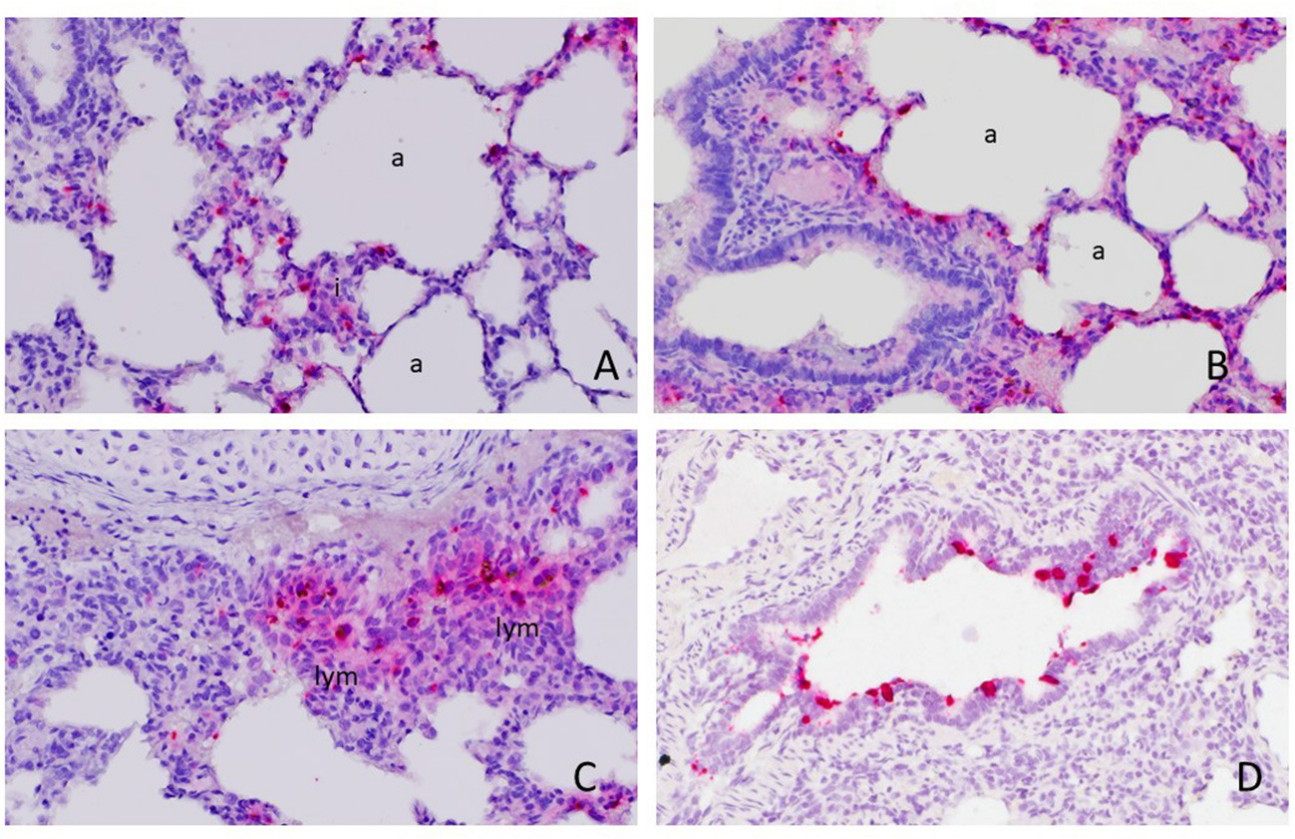
Detection of rotavirus A (RVA) nucleic acid in the lung by *in situ* hybridization (ISH). Positive red signals were detected in round cells resembling macrophages in the interstitium (i) or alveolar macrophages and pneumonocytes lining the alveoli (a) **(A)** and **(B)**. Focally, they were also detected in monocyte-macrophage lineage cells in peri-bronchial lymphoid aggregates (lym) **(C)** and bronchiolar epithelial cells **(D)**. Images were taken under 400x magnification.

Available (n = 22) whole tissue sections of RVA qRT-PCR-positive lungs were stained by ISH with a probe targeting the VP6 gene. Hybridization signals were observed in 4 of 22 cases. Intensive signals were detected in the cytoplasm of a few individual round cells with abundant cytoplasm, morphologically resembling alveolar and interstitial macrophages (Figure 1A). Occasionally, few pinpoint signals were also observed in cells with scant cytoplasm lining the alveoli or vascular capillaries, resembling pneumocytes or endothelial cells (Figure 1B). The hybridization signals were also detected in cells resembling monocyte-macrophage lineage cells in small peri-bronchial lymphoid aggregates (Figure 1C). In one case, there was focal, intensive ISH positive signals observed in a few bronchioles (Figure 1D), where approximately 20% of epithelial cells displayed ISH signals as red pinpoints in the cytoplasm or diffusely obscuring the entire cells.

### Detection of RVA in the Lungs Obtained From Pigs With Rotaviral Enteritis or Respiratory Disease by ISH and TMA

In the first TMA, RVA ISH positive signals were detected in 4 of 20 lung tissue cores obtained from piglets with rotaviral enteritis. Three cases had signals detected in cells resembling alveolar macrophages and pneumonocytes as described above. One case had intensive ISH positive signals in the bronchioles. An additional 6 cases with RVA ISH positive signals detected in alveolar macrophages were noted by screening the second TMA, which included 100 lung samples submitted for PRDC without information of RVA infection in the intestine.

### Validation of ISH Signals Detected in Bronchiolar Epithelial Cells

**Figure 2:**
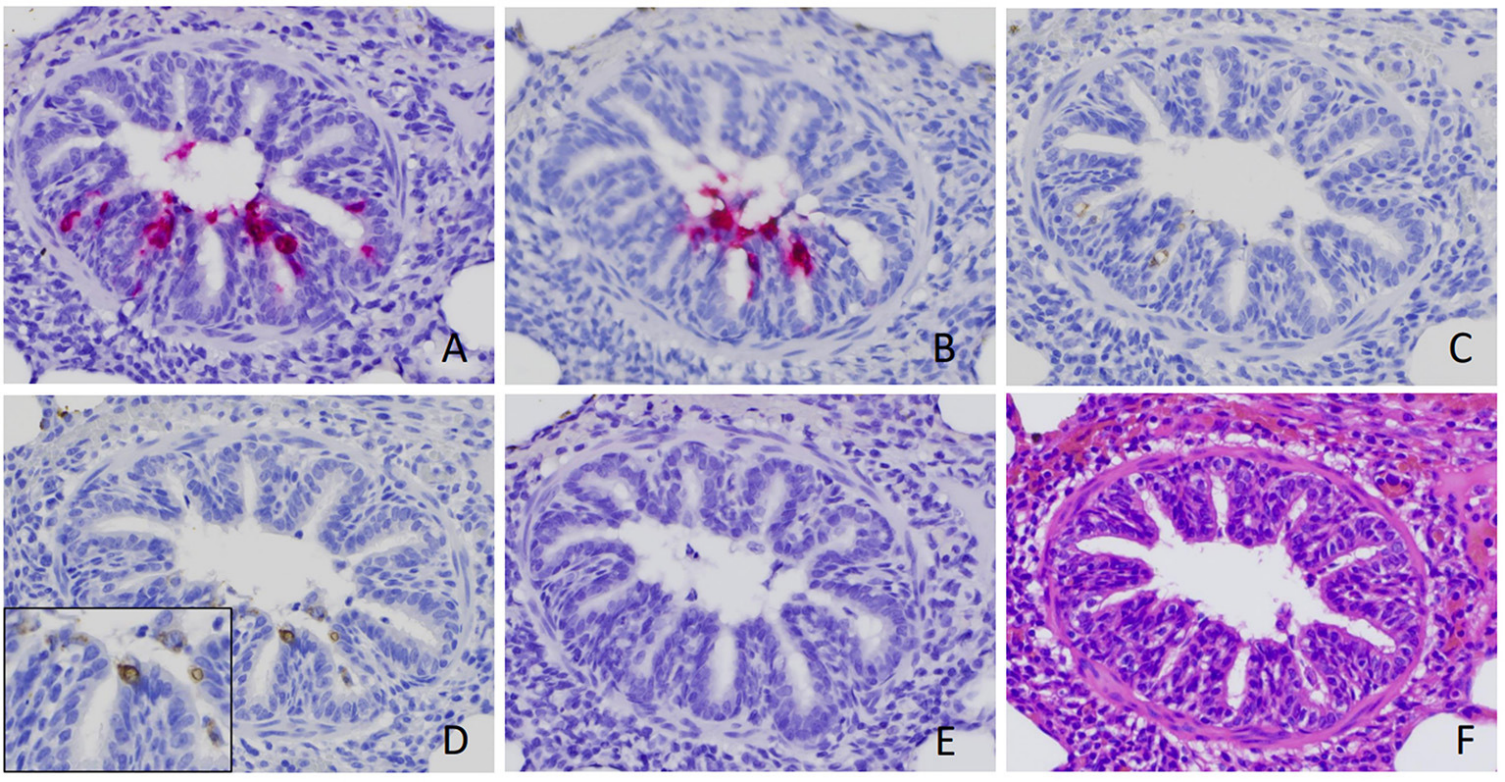
Verification of rotavirus A (RVA) infection in bronchiolar epithelial cells. Positive signal of RVA nucleic acid was detected by *in situ* hybridization (ISH) using probe targeting VP6 **(A)** and NSP3 **(B)**. Positive signals of immunohistochemistry staining using antibody against RVA VP6 **(C)** and dsRNA **(D)**, although weaker, were also detected in the serial sections obtained from the same case. No signal was detected by ISH using a probe targeting rotavirus B **(E)** (18). Serial section with routine hematoxylin and eosin stain **(F)** was performed. Images were taken under 400x magnification.

By using ISH with probes targeting RVA VP6 (Figures 1A, 2A) and NSP3 (Figure 2B), the same pattern of positive signal was detected in the same location in serial sections from the same case. Although fewer and weaker, positive signals were also detected in the bronchiolar epithelial cells by IHC with mAb against dsRNA and VP6 protein (Figures 2C,D).

### Visualization of Viral Particles in Bronchial Epithelial Cells by Transmission Electron Microscopy

**Figure 3:**
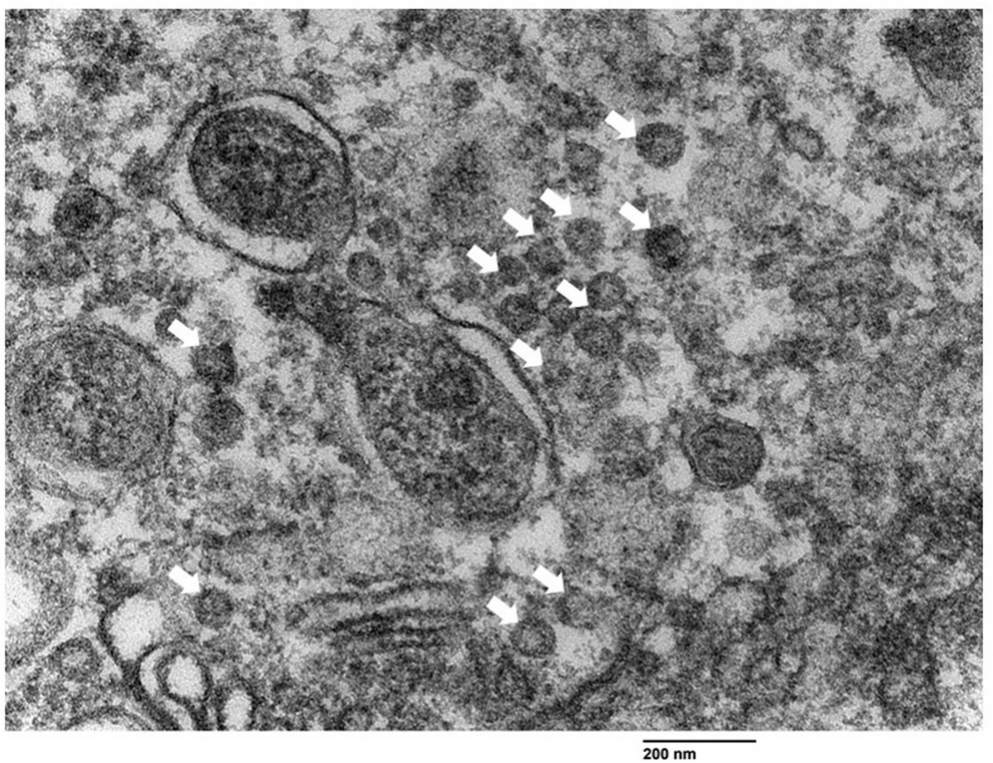
Visualization of rotavirus-like particles (VLP) in bronchial epithelial cells using transmission electron microscopy. VLP (arrows) ranging from 60 nm to 72 nm were observed near the apical border of the bronchiolar epithelial cells. Bar = 200 nm.

Particles measuring 60–72 nm in diameter compatible with RV, were observed inside rough endoplasmic reticulum vesicles or freely nearby the apical surface of bronchiolar epithelial cells (Figure 3).

## Discussion

Concurrent enteric and respiratory diseases can lead to significant losses in neonatal piglets. Data from the Swine Disease Reporting System consistently reports rotavirus as a leading pathogen identified in gastrointestinal disease submissions (21). Through our previous diagnostic testing of PRDC cases, unexpectedly, RVs were frequently detected in lungs using metagenomic sequencing, a diagnostic tool providing a comprehensive and unbiased identification of pathogens. This observation was supported by a high proportion of lung submissions that tested RVA positive by RT-qPCR in the present study. The positive cases are mainly, but not limited to, neonatal and nursing piglets. Given RV is not recognized as a primary respiratory pathogen in pigs, the high frequency of RVA detected in the lungs provided a justification for this investigation.

The pathogenesis of RV includes infection of non-dividing mature enterocytes, leading to cell death and malabsorptive diarrhea (22). Disruption of the intestinal mucosal barrier allows RV to gain access to the circulatory system as evidenced by observed antigenemia and viremia (5, 6, 23–25). An earlier study demonstrated gnotobiotic pigs inoculated intravenously with RVA developed fecal and nasal virus shedding, suggesting RV infection might be initiated from the basolateral side of the epithelial cells via viremia (6). In the present study, pigs with RVA qRT-PCR positive lungs had minimal or absent enteric signs based on reported clinical histories, although some diarrhea might be neglected clinically. In cases where both lung and intestine diagnostic results were available, RVA was detected in 8 out of 11 intestine samples from cases with RVA-positive lungs (Table 1). These results are consistent with a study in which RVA antigen was detected in 16 out of 58 cases (27.6%) of tracheal aspirates of children with a clinical diagnosis of pneumonia, only 2 of which had diarrhea with RV fecal shedding (26). Similarly, two studies reported RV detection in nasopharyngeal secretions from 28.1% (25 of 89) of infants or 8.9% (4 of 45) of children <5 years of age hospitalized for respiratory illnesses with or without diarrhea (8, 11). Experimentally, following oral or intranasal inoculation with attenuated human RV, 79% to 95% of gnotobiotic pigs developed nasal shedding of infectious viruses, while only 5% to 17% shed virus rectally (6). Overall, these results suggest that respiratory and enteric RV infections can be independent events, although they frequently occurred in the same cases.

In the present study, two sample selection strategies were used to investigate the infection of RVs in the lungs. Among 22 RVA qRT-PCR positive cases submitted for PRDC diagnosis with FFPE tissues available, 4 cases were tested ISH positive in whole FFPE lung sections. Likewise, RVA-positive signals were detected in 4 of 20 TMA cores obtained from piglets with high titers of RVA detected in the intestine from archived samples. Regardless of the different criteria and strategies used for case selection, these approaches resulted in no obvious differences in the detection rate of RVA in respiratory tissues.

In addition to mature enterocytes in the small intestine, previous studies indicated a broad cellular tropism of RV infection (5, 6). In the present study, signals of the RVA genome and antigen were detected in macrophages and bronchiolar epithelial cells, as well as leukocytes and endothelial cells in vascular capillaries, and cells resembling the pneumonocytes in the lungs. Detection of RV antigens in alveolar macrophages has been described, however, the pathologic relevance is undetermined (9, 10). Alveolar and interstitial macrophages in the pulmonary reticuloendothelial system function to clear pathogens from the bloodstream (27). Intensive RVA ISH and IHC signals in the macrophages could result from phagocytosis and accumulation of rotaviral materials filtered through the bloodstream, whereas detection of RV in other cell types suggests infection. In humans, three separate fatal cases classified as pneumonitis and acute interstitial pneumonia showed rotaviral RNA localized in alveolar capillaries, macrophages, and pneumocytes by *in situ* RT-PCR (9, 10). Likewise, inconspicuous ISH signals of RVA genomes were sporadically noted in a few individual cells lining the alveoli and vascular capillaries in the present study. Weak ISH signals reflect that localization of low levels of extra-intestinal RV infection is limited by the sensitivity of tissue-based assays as compared to qRT-PCR. Resende et al. (18) also noted some RV PCR-positive samples that were negative by ISH possibly explained by uneven virus distribution throughout tissues or the presence of multiple RV subtype that did not cause lesions (18). To account for genetic variability, we designed a RVA probe targeting a second RV gene, NSP3, and performed IHC with anti-dsRNA. Tissue sections stained similarly, regardless of method, thus indicating genetic variability was not the sole cause of decreased sensitivity in ISH and IHC assays as compared to qRT-PCR.

Intensive ISH and IHC RVA signals detected in bronchiolar epithelial cells here are evidence of infection. Visualization of viral-like particles by TEM suggested the virus could complete its replication cycle. To the best of our knowledge, the infection of RV in bronchial epithelial cells has not been reported elsewhere. Rotavirus replication in the respiratory epithelium is consistent with reported nasal RV shedding (6).

An intensive ISH signal of RVA in bronchial epithelium was detected in two cases. Although the specificity of ISH was verified by different probes and supported by IHC and TEM, many questions regarding this finding remain unanswered. Unlike the typical widespread pattern of RVA infection in the small intestines, the hybridization signal of the RVA genome was only observed in a few bronchioles in a small subset of pigs in this study. This observation might be explained by the difference in anatomy and physiology between the digestive and respiratory tracts. The branching architecture of airways can limit the spread of the virus in the lung. Although RV may reach the bronchiolar epithelium by viremia, direct contact of the airway surfaces, through aspiration of RV-contaminated fecal materials or aerosols in the farm, may better explain the focal or multifocal pattern of RVA ISH signals observed under the microscope. Further research on possible aerosol transmission of rotavirus to pigs exposed to high environmental burdens is warranted. In addition, RV antigen was detected in the lung of one of 13 experimentally infected 3-week-old conventional pigs, suggesting individual variation can play a role (6).

The role of RVA genetics on respiratory tropism is unknown. Azevedo et al. (6) demonstrated that gnotobiotic piglets inoculated with virulent and attenuated RVA resulted in different nasal and fecal shedding patterns that they hypothesized that lower temperature in the respiratory tract may facilitate attenuated RV infection (6). In the present study with samples collected from the field, coinfection of pigs with several different RV strains, and multiple bacterial and viral co-infections in the same lung sample, limited our ability to assemble RVA genome sequences directly from lung specimens using metagenomic sequencing assays. Targeted sequencing of rotavirus in enteric and respiratory tissue would be useful for investigation of genetic markers of rotavirus tissue tropism. These sequences would likewise be valuable for the design of diagnostic tests with increased sensitivity. Cell damage and disruption of the respiratory mucosa are thought to promote secondary bacterial infection. Whether RVA infection can provide the initial viral insult that leads to secondary bacterial pneumonia is still unknown. The limited dataset presented here does not provide clear evidence of a correlation between rotavirus and respiratory disease. Further experimental animal studies are merited.

Awareness of extra-intestinal infection and systemic impacts of RV is increasing (5–11, 23, 28–30). Classically known respiratory or enteric infections frequently cause multisystemic disease (31). Here we demonstrated a high prevalence of RV detected in the lungs obtained from conventional neonatal and nursing piglets with respiratory signs, which is similar to earlier observations in infants and children (28–30). These observations were supported by the localization of the RVA genome, antigen, and viral particles in the bronchial epithelial cells. Further work is needed to elucidate the clinical significance of RV respiratory infections.

## Data Availability Statement

The raw data supporting the conclusions of this article will be made available by the authors, without undue reservation.

## Ethics Statement

Ethical review and approval was not required for the animal study because the porcine samples used in this study were collected by veterinarians as part of their professional care of client animals and submitted for diagnostic testing. As such, no specific IACUC approval was required. Written informed consent for participation was not obtained from the owners because consent for diagnostic testing is given by submission of samples for diagnostic testing.

## Author Contributions

AN: investigation and writing-original. C-ML: conceptualization, methodology, formal analysis, writing-original and review & editing, supervision, and funding acquisition. BH: conceptualization, methodology, writing-review & editing, supervision, and funding acquisition. KL: writing-review & editing. EN: writing-review and editing and funding acquisition. JS: investigation, writing-original, formal analysis, and methodology. All authors contributed to the article and approved the submitted version.

## Funding

This research was in part funded by American Association of Swine Veterinarians (AASV) Foundation and supported by the Animal Disease Research and Diagnostic Laboratory at South Dakota State University and USDA Hatch funds.

## Conflict of Interest

The authors declare that the research was conducted in the absence of any commercial or financial relationships that could be construed as a potential conflict of interest.

## Publisher's Note

All claims expressed in this article are solely those of the authors and do not necessarily represent those of their affiliated organizations, or those of the publisher, the editors and the reviewers. Any product that may be evaluated in this article, or claim that may be made by its manufacturer, is not guaranteed or endorsed by the publisher.
